# Flexible Tactile Sensor System Based on Piezoresistive Layer: Technology and Construction

**DOI:** 10.3390/s26113345

**Published:** 2026-05-25

**Authors:** Viktor Novák, Jaromír Volf, Roman Hrmo, Petra Kvasnová, Vladimír Ryženko, Daniel Novák, Alena Očkajová

**Affiliations:** 1 Department of Electrical Engineering and Automation, Czech University of Life Sciences Prague, 165 00 Prague, Czech Republic; volf@tf.czu.cz (J.V.); ryzhenko@tf.czu.cz (V.R.); 2 Department of Didactics of Technical Subjects, DTI University, 018 41 Dubnica nad Váhom, Slovakia; hrmo@ivp.czu.cz; 3 Department of Technology, Matej Bel University, 974 01 Banská Bystrica, Slovakia; petra.kvasnova@umb.sk (P.K.); daniel.novak@umb.sk (D.N.); alena.ockajova@umb.sk (A.O.)

**Keywords:** pressure, sensor, piezoresistive, tactile system, conductive ink

## Abstract

**Highlights:**

**What are the main findings?**

- Custom blended conductive inks are suitable to act as a transducer between applied pressure and electrical resistance.

**What are the implications of the main findings?**

- On the basis of the piezoresistive effect of conductive inks, an original integrated tactile system, SITSCAN CS, was developed.

**Abstract:**

SITSCAN CS is an original tactile system, which was primarily developed to investigate pressure distribution on uneven surfaces, e.g., chairs; however, due to its flexibility and modular conception, it can be utilized in other industrial or medical applications too. It consists of a flexible, PET-based PCB print-made sensing plate with active area of 50 × 50 cm with a placed matrix of 50 × 50 individual sensors. It uses the piezoresistive effect of the conductive ink layer as the transducing technology between the applied pressure and the output electrical signal. The tactile system further consists of control electronic circuits which process the measured data with up to 1000 fps with a maximal possible resolution 80 × 80 sensing points. The acquired data can be visualized, stored and further processed by means of the respective PC control program. The article describes the theoretical basis for the tactile system, as well as its development, construction, technical specifications and the testing process.

## 1. Introduction

### 1.1. Theory and Design of Planar Pressure Transducers

Planar pressure transducers, also called foil transducers, convert the applied pressure over an area into electric signal, so they are able to create so-called pressure maps. When pressure is applied, the material of the transducer is deformed and it changes its mechanical, electrical or other physical properties, which is evaluated by a respective measuring element. There are various principles that determine how to evaluate the degree of deformation of the material: change of electrical resistance, change of capacity, change of magnetic properties of the transducer material, piezoelectric principle, triboelectric principle and change of optical properties of the transducer are the most used technical principles of planar pressure transducers. Based on the stated technologies, there have been a lot of recently developed pressure transducers using modern polymer materials, used primarily in robotics, medicine or as artificial skin [1,2,3,4,5,6,7].

Each of the stated technologies has its advantages and disadvantages, primarily the level of sensitivity and the measuring range, the level of complexity of the measuring electronic circuits, mechanical endurance, the level of flexibility to adopt uneven surfaces, possible temperature dependency of the sensor and others.

The planar pressure transducer can be designed as an array of individual sensing elements, where individual pressure level (relative or absolute) is assigned to each sensing element. The arrangement of the sensing elements depends on the proposed use, size and shape of the sensing area; possible variants are, e.g., hexagonal, triangular or matrix array [8,9].

The potential use of planar pressure transducers is very varied: in the automotive industry by designing car seats, by designing car, truck or tractor tyres, in agriculture by analysis of soil compaction, in the design of ergonomically shaped furniture or in medicine by analyzing the foot imprints or other body contact pressures.

### 1.2. The Piezoresistive Technology and Transducers

**Figure 1:**
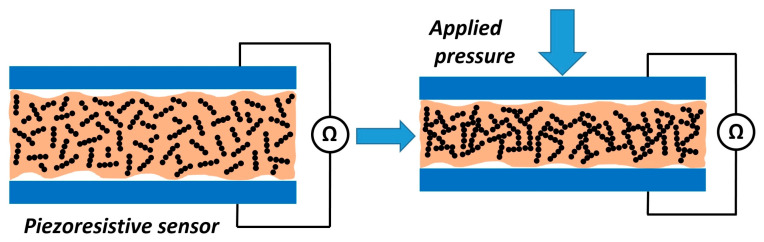
The principle of a piezoresistive sensor. (**Left**)—without applied pressure, (**Right**)—maximal compression and saturation.

Considering the possible technical properties and requirements on using various transducing technologies (particularly the predicted working range, technology of manufacturing, level of flexibility, and technical solution of the electrical control circuits), we have decided to use the piezoresistive technology in sensor system. The original name comes from the Greek language (“piezin” = “to press”), where the electrical resistance of a conductive layer changes under the applied pressure (Figure 1), according to the Formula (1); however, this formula is only valid in a limited pressure range (linear range, usually the working range of the transducer).

dR/R = K × ε(1)

where 

R [Ω]—Electrical resistance of the unstrained material.

dR [Ω]—Difference of the electrical resistance of the pressed material.

K [-]—Gauge factor.

ε [-]—Mechanical strain.

As pressure is applied, the microscopic conductive particles (so called filler) in the transducer approach one another and this causes a decrease in the measured electrical resistance of the material. The value of the electrical resistance is subsequently used to compute the pressure. Exceeding a specific pressure, all the conductive particles are connected to each other, so there are no more conductive canals created. Thus, the conductivity of the material increases then just slightly or not at all. This phenomenon is called saturation (Figure 1—right); it strongly depends on the used piezoresistive material, and it is one of the main factors that defines the usable measuring range. An extensive theoretical review about the piezoresistive effect and its application in sensor systems can be found in [10,11].

Piezoresistive planar pressure transducers originally used conductive rubber, based on the experiences described by Barman and Guha [12]. Afterwards, we focused our research on creating a flexible transducer, and we reflected newer experiences with different piezoresistive materials, e.g., the design of FSR sensors discussed in [13] with lower pressure range. A similar solution using printing technology used in robotics is described in [14]; however, they only produce a binary output signal which is not usable for our requirements. Another flexible pressure sensor is being developed by Maddipatla et al. [15], and they use different technology (capacitive sensors) with promising results; however, its sensitivity would be insufficient for our purposes. Another interesting solution using a paper-based piezoresistive, highly sensitive pressure sensor is described in [16]; however, this technology has limitations due to possible mechanical wear of the material. A flexible pressure sensor using a tactile sensing field on a board made by PCB technology was first presented by Castellanos-Ramos et al. [17]. It came originally with a matrix of 20 × 20 electrodes printed on PET foil; PEDOT conductive ink was used as the piezoelectric material. A simple sensor designed directly to measure the pressure distribution on the seat of a wheelchair chair was developed by Ahmad et al. [18]. This sensor consists of 16 sensing elements, uses conductive ink with carbon and silver microparticles and serves primarily for long-term monitoring of the position of a seated person; from this point of view, the small spatial resolution of this sensor is sufficient. A review of the most recent use of flexible tactile sensors, primarily in the field of biomechanics, can be found, e.g., in [19].

On the market, there are already available tactile systems that enable pressure distribution measurements on uneven surfaces, e.g., chairs. The main products are Tekscan BPMS (Body Pressure Measurement Systems) [20], XSENZOR ForeSiteSS [21] and Novel Pilance [22]. The named tactile systems use piezoresistive or capacitive transducing technology with resolution up to 4 sensors/cm^2^, maximal sample frequency 50 fps and pressure range up to cca. 100 kPa. Our proposed tactile system should equal the commercially available systems in some parameters.

In our previous work, we developed a planar measuring system PLANTOGRAF which was primarily designed to measure foot imprints in medicine, but it found its use in the automotive industry to measure pressure distribution between road or soil and tyre too. Its current version V18 consists of a matrix of 1600 individual sensors that change their electrical resistance due to the applied pressure. The Plantograf and its predecessors used different technologies: originally conductive rubber, and further conductive ink on a separate foil layer; for more detailed information about the previous research, see [23,24]. However, the use of this sensor was limited to evaluate the contact pressures on even surfaces, and due to the quick recent development of new, flexible materials and new microcontrollers with better resolution, sampling frequency and connectivity, the measuring system became obsolete. Further negative aspects were substantial hystheresis of the originally used conductive rubber as well as the mutual interaction of the neighboring electrodes, as they were sharing a common piezoresistive layer; with the new design, we would like to eliminate these negative aspects, too.

Our goal is to develop a new, flexible measuring system named SITSCAN CS, which will be able to adapt to uneven surfaces, and which is primarily designed to measure the pressure distribution on chairs. The main motivation was to create a tool that helps to develop economically shaped school chairs to increase the efficiency of the educational process. This brings some innovative techniques of the design, such as using the PCB printing technology by creating the sensor matrix and using the newest microprocessor technology to allow sufficient performance margin.

## 2. Materials and Methods

### 2.1. Tactile System Requirements

**Figure 2:**
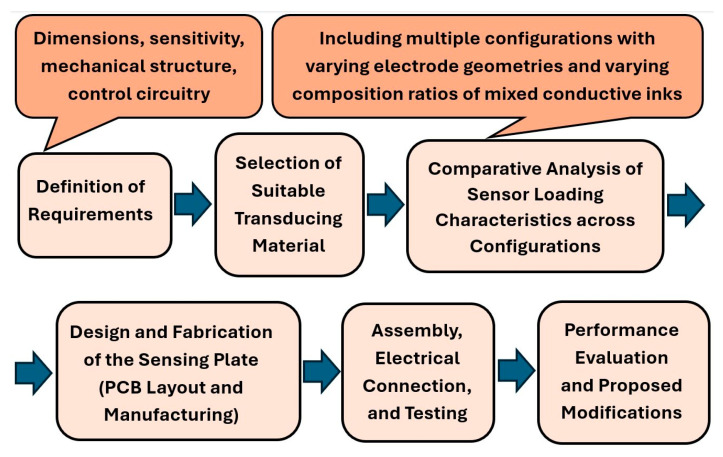
Sketch of the manufacturing process.

The proposed tactile system SITSCAN CS will be primarily intended to measure the pressure distribution on uneven surfaces, such as chair seats, on a relative 8-bit scale. Its design should be able to primarily capture excessive contact pressure areas on the investigated surfaces that could cause medical difficulties or mechanical wear. The design will consist of a sensing plate 50 × 50 cm, with a matrix configuration of the sensing elements with a suggested spatial resolution of 1 sensor per 1 cm^2^. According to the preliminary calculations based on the pressure of the pelvic bones (estimated by cca. 120 kPa), the measuring pressure range should be between 30 and 200 kPa, so that areas of lower pressures can be captured, and mainly the possible peak pressure values too. Further parts of the measuring system will be electronic circuits that control the data acquisition from the sensor matrix and that convert the acting pressure into electric signal as well as a PC program that saves, displays and analyses the measured data. The sketch of the proposed manufacturing process is stated in Figure 2.

### 2.2. Measuring of the Piezoresistive Layer

As we discussed above, we have selected the piezoresistive technology for the proposed tactile system. The main advantages were relatively simpler electronic control circuits and previous experience with this technology within our existing research. We have also decided to use conductive ink as the transducing material between the applied pressure and electrical quantities. Unlike the conductive rubber, used previously in the planar pressure transducers, conductive ink exhibits almost no hysteresis, the sensor can be made using the PCB printing technology and the use of the ink makes it possible to select the desired sensitivity of the ink layer by blending conductive and non-conductive inks together; an overview about the use and properties of conductive inks can be found in [25].

The selection of the piezoresistive layer depends on the mechanical and electrical properties of the materials; we considered several conductive inks (e.g., KH WS SWCNT; Luxor; NGAP FI Ag-4101 and DZT-3K). The first two water-based inks were not usable because they could not create a coherent layer. The NGAP ink exhibited excessive conductivity in the range of units of Ω. The DZT-3K ink exhibited better mechanical and electrical parameters; however, it could not be attached to the electrodes directly, but only in form of a foil layer, which we refused due to requirements regarding the manufacturing technology.

After further investigation, Loctite NCI 7002 and Loclite ECI 7004HR inks (manufacturer Henkel, Stamford, CT, USA) were finally selected, which are mechanically intermixable. These inks are made on a polyester and polyamide basis, with NCI 7002 ink being non-conductive and ECI 7004HR ink being conductive with graphite microparticles as filler; both inks are intended for the creation of mixtures to achieve the desired electrical conductivity, and they are suitable for printing technology too. Given the polymer base of the inks, better integrity of the ink layer and better adhesion to the electrode can be expected. The electrical resistance value of the unloaded layer of the mixture of both inks in a 50:50 ratio is 96 kΩ·mm^−2^; the datasheets of the inks are stated in [26,27].

**Figure 3:**
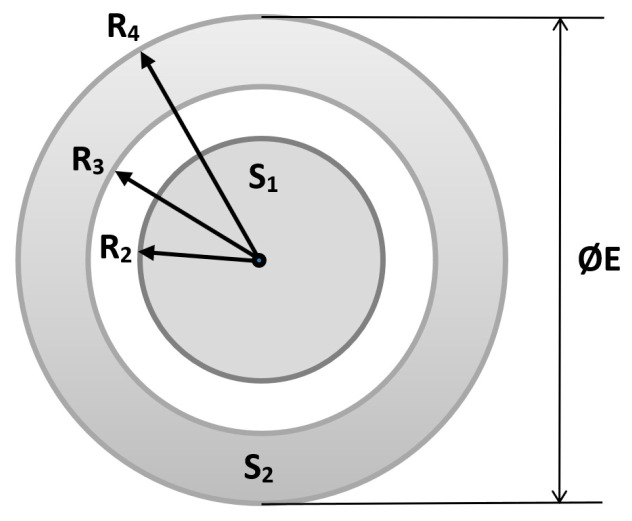
Design of the electrodes.

Because there was neither experience nor data of the transducing characteristics of a piezoresistive layer created by conductive ink blends, we had to determine them experimentally. The original methodology of the measurements of electrical properties of piezoresistive materials is based on the experiments of Souza et al. [28]; we have further modified it and used it in our previous research [29]. A total of 18 individual electrodes with different dimensions were placed on a “plate” with a specified ink layer; there were, in total, 162 individual samples to measure (18 electrode dimensions, 3 ink layers and 3 ink mixtures). The technical properties of individual variants (ink layer properties) are listed in Table 1. The shape and dimensions of the electrodes are depicted in Figure 3. The electrodes adhere to the piezoresistive layer created by the conductive ink blend; the cross-section and the overall design of the sensing plate are described further in Chapter 2.3.

**Figure 4:**
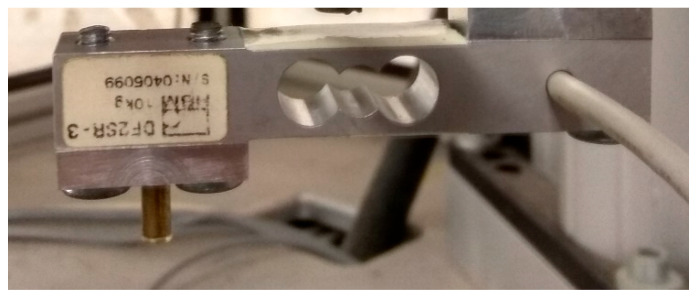
Hottinger DF2S-3 load cell and the measuring tip.

Due to the extent of the performed measurements of individual pressure-resistance characteristics, this procedure was partially automatized using a robotized workspace to obtain the required pressure in all points of the loading characteristics. The loading was controlled using the Turbo Scara SR60 (manufactured by Robert Bosch Robotics GmbH, Renningen, Germany) robot with an attached measuring tip connected to a Hottinger DF2S-3 load cell (manufactured by Hottinger Baldwin Messtechnik GmbH, Darmstadt, Germany) at the end of the robot’s arm (Figure 4). Pressure was applied by the measuring tip, 3 mm in diameter, by means of the vertical motion of the robot’s arm. The arm was moved in 0.02 mm increments (loading only), for a general overview of the loading characteristics, and further in 0.01 mm steps (both loading and unloading) for a more detailed analysis; this more detailed course was measured only on selected sensors. The loading force was exerted from 0.37 N up to cca. 17.6 N. The applied pressure was calculated from the known area of the surface of the measuring tip and the exerted force. This resulted in the measured range of pressure values approx. from 30 kPa up to 1400 kPa for the particular measuring tip.

**Figure 5:**
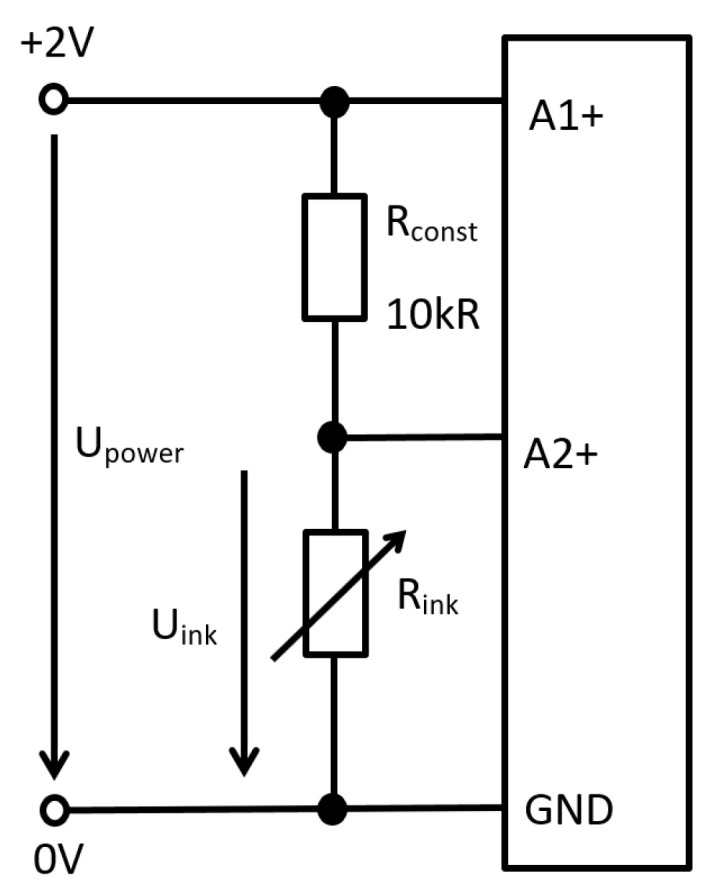
Simplified circuit diagram.

Each one measurement for the selected acting load was repeated 10× and the data was filtered (removing lowest and highest value from the data set). The loading characteristics of the individual sensors were measured in the connection as the voltage divider, where the captured output value was the voltage between A2 and GND, as depicted in the simplified circuit diagram in Figure 5. This setup was chosen because it corresponds to the proposed circuits of the entire sensing plate. The measured voltage was subsequently recalculated to electrical resistance.

**Figure 6:**
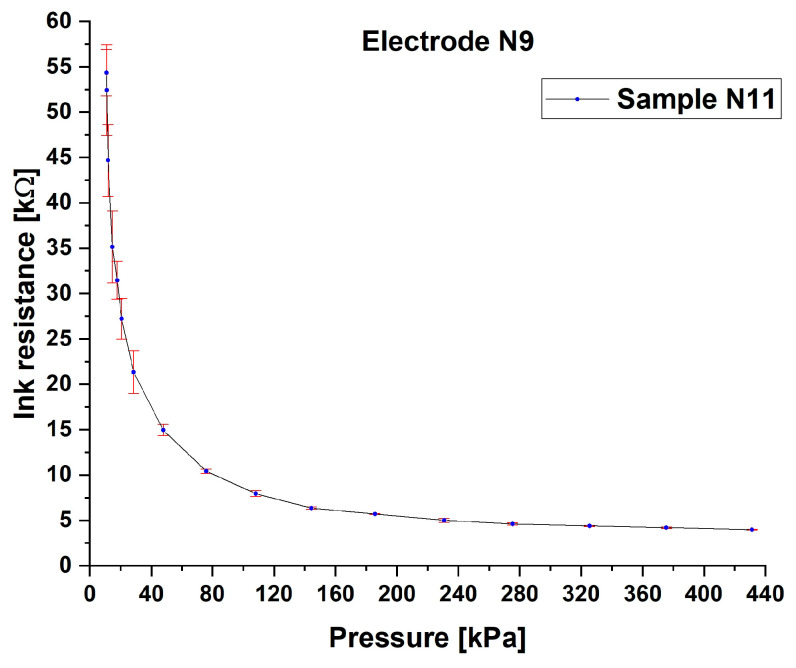
Loading characteristics; dependency of the electrical resistance on the applied pressure. Sample Nr. 11, Electrode Nr. 9.

After the evaluation of all 162 measured characteristics, the finally selected sample was Nr. 11 placed with Electrode Nr. 9, of which technical parameters are as follows; the loading characteristic of this single sensor is depicted in Figure 6.

- Electrode dimension: circular, outer diameter 1.75 mm.
- Ink composition: HENKEL NCI 7002 and ECI 7004HR in ratio 40:60.
- Ink thickness: 24–27 µm.

In general, the sensor loading characteristic exhibits an initial steep decrease in electrical resistance followed by a turn when the resistance decreases significantly more slowly with the rising pressure. This turn is situated in the pressure range 200–400 kPa, depending on the electrode size and ink composition; also, the turn is differently sharp. This phenomenon is caused due to the exponential dependency of the resistance on the pressure, which is based on the composition of the material; as the pressure is high enough, there are created significantly fewer additional conductive paths, and thus, the resistance drops only a little. However, the saturation of the material occurs significantly above the proposed measuring range of the transducer.

Further, the uncertainties are generally much greater in the range of lower pressures, particularly in pressure ranges under the described turn. This is given first by the light contact of the measuring tip with the surface and secondly by the light contact of the conductive layer itself with the electrode.

During the loading and unloading cycle, no measurable hystheresis was detected; the possible drift was not investigated given the relatively short period of time assigned to individual loading level (cca 10 s). According to the intended use of the tactile sensor, while a person sits on the chair for a short period of time, this would not have any negative impact on the results. The same is valid for possible temperature effects; given the intended use of the sensor, they are not expected to influence the resulting imprint. The sensor should measure relatively brief contacts under stable conditions on a relative scale. If there would be a negligible impact of temperature shift or drift, it would not negatively affect the pressure map, where primarily excessive pressures are investigated.

The selected sample exhibited stable loading characteristics (without excessive fluctuations) and the saturation of the material after the value of the applied pressure of 200 kPa; other samples exhibited either very early saturation of the piezoresistive layer or they were loaded with excessive fluctuation or uncertainties. This particular sensor specification was subsequently used for the manufacturing of the prototype of the SITSCAN tactile system.

### 2.3. The Sensing Plate

**Figure 7:**
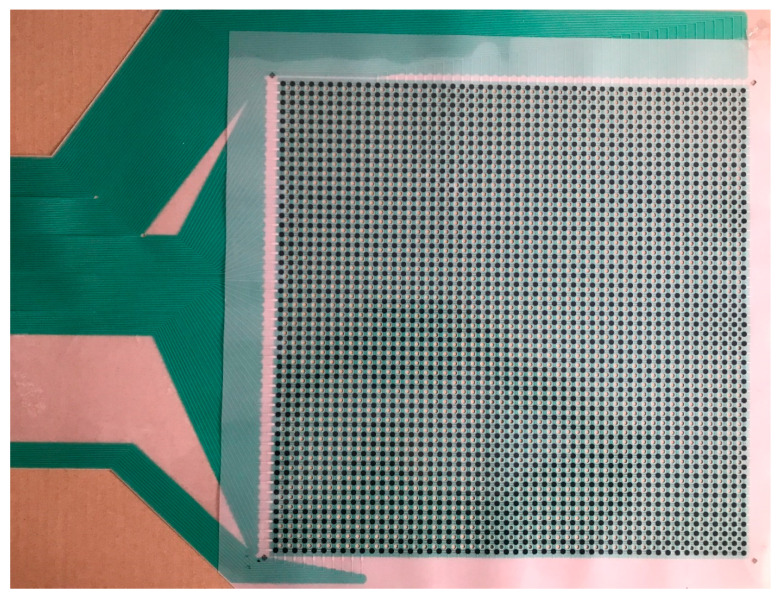
The sensing plate of the SITSCAN CS tactile system.

**Figure 8:**
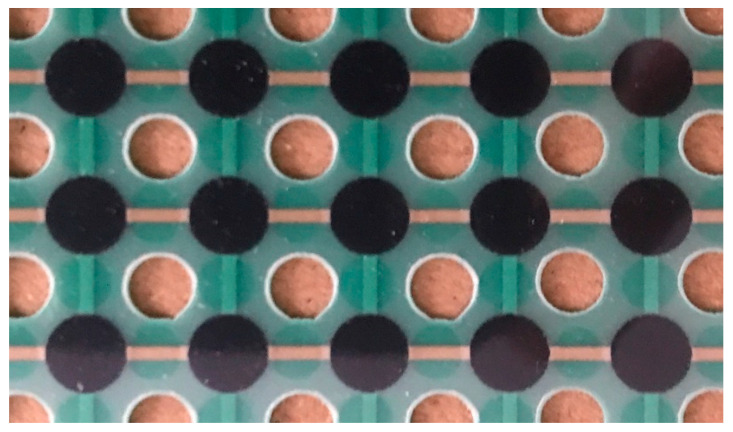
Detail of the sensing plate of the SITSCAN CS tactile system.

According to the requirements of the assignment, the entire structure is designed to be flexible (unlike previous sensors developed within our previous research), i.e., it will be able to adapt to uneven surfaces, such as the seat of a chair. Thus, we have selected a flexible and PCB-capable support for the electrodes’ matrix −150 μm thick PET foil. The sensing plate (Figure 7) has an active area of 50 × 50 cm, which will cover the most chair seats. The sensor array was designed as a matrix, mainly for even distribution across the area, for the requirements of the PCB print and for operational reasons of the electronic control circuits. For reasons of feasibility of the sensor design, the original arrangement of 80 × 80 sensors to 50 × 50 sensors has been abandoned. Due to the denser arrangement of electrodes, the original concept did not achieve the required elasticity necessary for measuring uneven surfaces and was prone to fractures and cracks. With the originally considered resolution of the sensing matrix, it would not be possible to place holes in the structure, which serves to increase the elasticity of the sensor and reduce the occurrence of fractures. These holes are always located in the middle of the square, the tops of which are formed by individual electrodes (black points); see the detailed structure of the sensing plate in Figure 8.

Given the dimensions of the sensing plate, it corresponds to the sensing matrix resolution of 1 sensor per 1 cm^2^. In total, there will be 2500 sensors on the sensing plate— individual electrodes. The measuring range will be primarily set for the intended use, i.e., for measuring contact pressures between the chair seat and the seated person; the considered range is set at 30–200 kPa according to the indicative calculation; if necessary, the signal can be amplified via the control program too.

**Figure 9:**
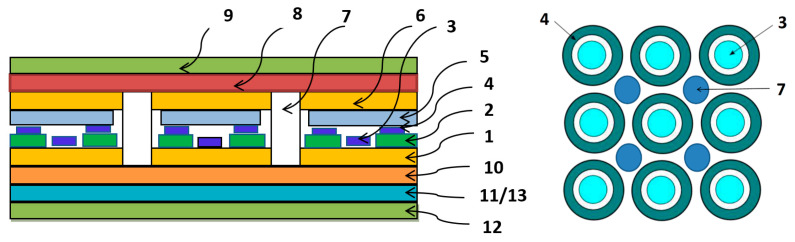
Cross section and top view on the sensing plate.

The cross section and the top view on the sensing plate of the sensor are depicted in Figure 9 left. The sensor is a sandwich construction with several layers. Each tactile sensor consists of an elastic, non-conductive carrier foil (1), on which conductive ink is applied. The inner ring electrodes (3) of the sensor are applied to the elastic carrier film (6), followed by a dielectric layer (2) with holes in the places of the inner ring electrodes. The outer circular electrodes (4) are printed on this dielectric layer. The electrodes touch the ink layer (5). These layers are covered from both the bottom and the top with an elastic protective non-conductive top layer (9/12) and they are supported by a solid box (11/13). A slide layer (8) optimizes the pressure distribution to the electrodes. Circular holes (7) can be placed in the space between the electrodes to improve elasticity and to avoid the spread of possible cracks.

### 2.4. The Electronic Control Circuits

The electronic circuits of the sensor are used to evaluate and process the data of individual rows and columns of the tactile matrix. Specifically, the electronic circuits of the sensor perform following functions:

- **Selector**: A MOSFET-controlled active part matrix selection unit; it selects the row to be evaluated by switching voltage to that row.
- 4× MCU: 4× **matrix operation block**, where each of the blocks covers 20 columns of the matrix.
- 1× MCU: **data aggregation block** for control, timing and processing of data from individual matrix operation blocks and communication with superior layers (PCs, data storages).
- **Power supply and auxiliary circuits**.

**Figure 10:**
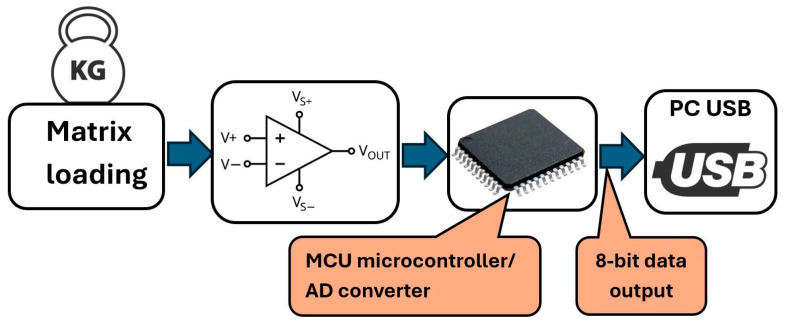
Block diagram of the tactile sensing system.

A block diagram of data acquisition and processing is depicted in Figure 10.

**Figure 11:**
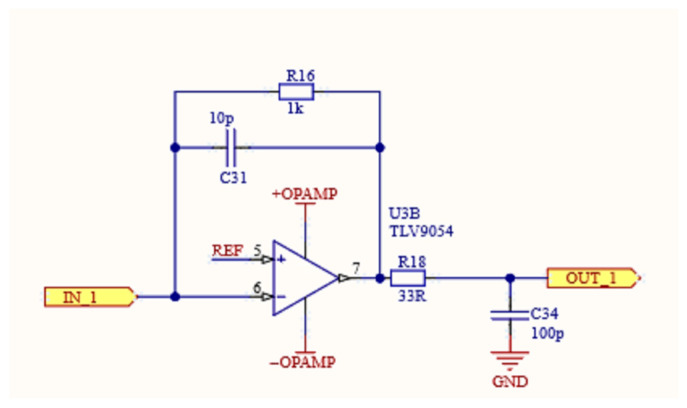
Pre-processing of the signal using OA, input IN_1; output OUT_1.

The raw data from the matrix of electrodes are obtained in the form of the voltage level from the volage divider that creates the piezoresistive layer (see circuit diagram in Figure 5 above). This value is further pre-processed by OA (Figure 11) to amplify and partially linearize the signal; this modified output is subsequently processed in the ADC within the microprocessors. The built-in ADC converts the analog signal into 8-bit digital output that is further processed by another MCU, which controls the timing and the communication with the PC control program.

**Figure 12:**
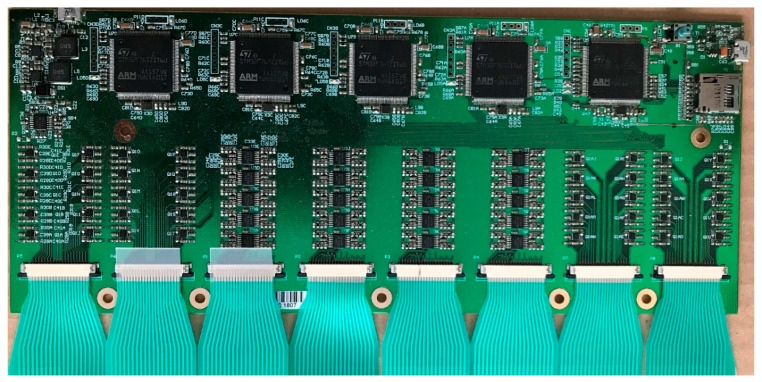
The control electronics of the SITSCAN CS tactile system.

The core of the control electronic is the STM32F767ZIT6T processor, with maximum core frequency up to 216 MHz. An 80-channel ADC MCU was not available, so we decided to use 4 identical 20-channel MCUs. It contains 12-bit ADCs to process the analog signal from the individual sensors in parallel operation, with a sampling rate up to 2 MSps [30]. One MCU processes 20 columns of the electrode matrix, and the evaluated rows are switched using the selector as described above. At maximum speed, data can be taken from the whole matrix in 280 μs, which is a time fully sufficient for the considered frame rate of up to 1000 Hz. The power supply levels are 3.3 V and 5 V; the lower value is used to power the microprocessors, ADCs and the sensing matrix, while the 5 V level is used for the USB interface. The printed circuit board with installed components is depicted in Figure 12.

### 2.5. The PC Control Program

**Figure 13:**
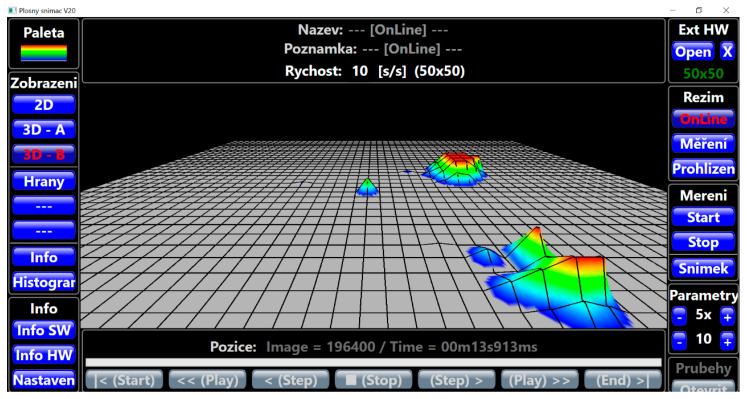
Main screen of the SITSCAN CS PC control program in 3D view.

The PC control program includes basic tools for capturing, storing, and displaying data, with expansion options for further analysis. The program allows you to save individual images and record video sequences. The pressure on the pad is displayed in the color scale: gray (without measurable load)–blue–green–yellow–red from the lowest to the highest load in 256 levels; hence, only an 8-bit range of the ADCs is used. The red color (value of 255 AD converter) represents the maximum pressure value in the given range; in calibration measurements at optimal gain and offset settings, it represents a pressure above 120 kPa. By selecting the appropriate amplification, this level can be shifted to highlight the peak pressure values in the measured range. The screen of the control program is depicted in Figure 13. The control program enables, among other things, the following functionalities:

- Possibility of mirroring the image from the sensor along the X and/or Y axis.
- Function to view the sensing area “from below”.
- 3D display. The pressure is displayed not only on a color scale, but also spatially in the form of elevations above a flat surface.
- Possibility to save the image to a file as a regular image.
- Extension of the number of adjustable gains.
- Storing data in a database.

**Figure 14:**
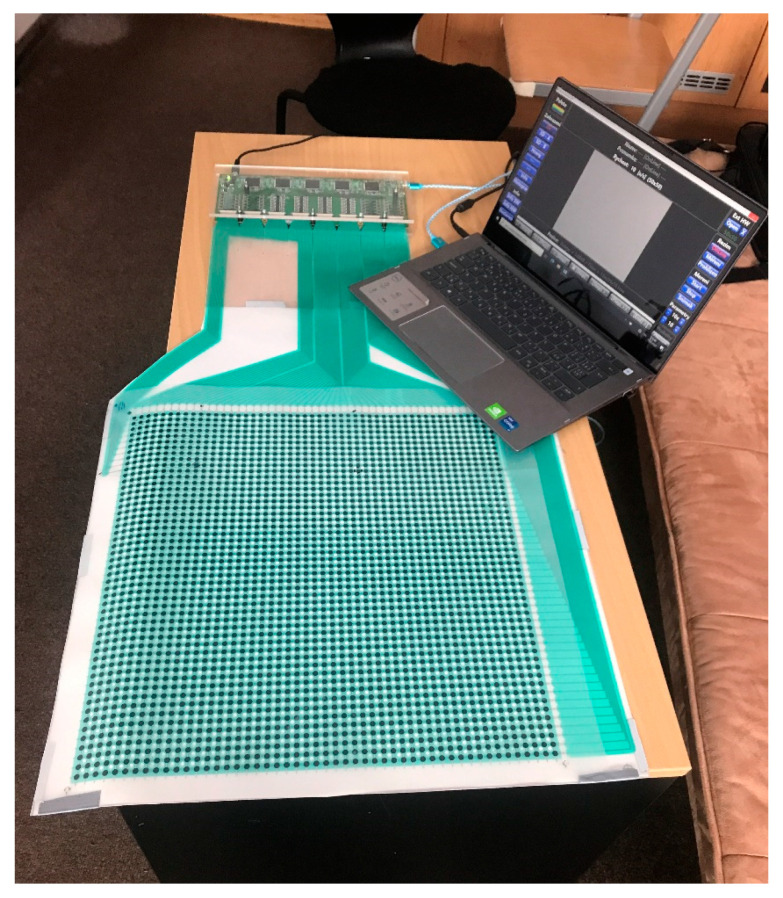
The complete SITSCAN CS tactile system.

The view on the complete SITSCAN CS measuring system, i.e., the sensing plate, control electronics and a connected laptop, is depicted in Figure 14.

## 3. Results

**Figure 15:**
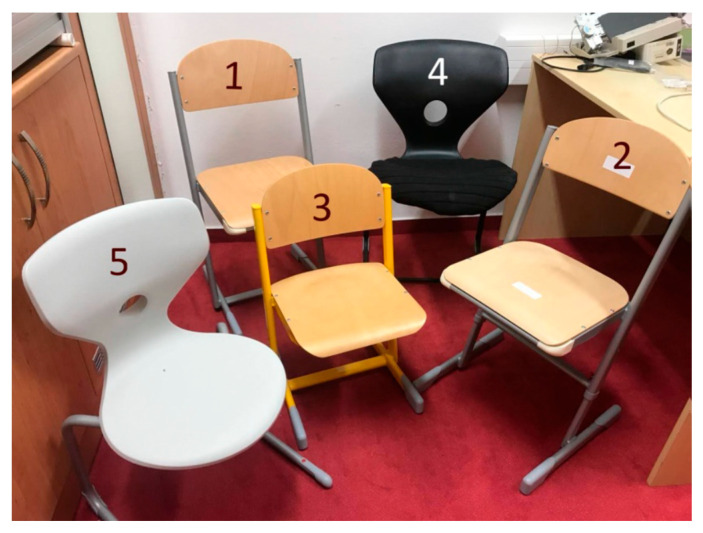
Tested school chairs.

The SITSCAN CS tactile system has been subsequently tested to see if it meets the specified requirements. As part of the initial testing measurement, we compared the properties on five different chairs; see Figure 15. The chairs are a product of the company Santal (Trebon, Czech Republic), which designs and manufactories school furniture. The SITSCAN CS tactile system will undergo testing within this company. For comparison, we measured the pressure distribution of a sitting person (60 kg) on all five types of chairs.

### 3.1. Initial Testing

**Figure 16:**
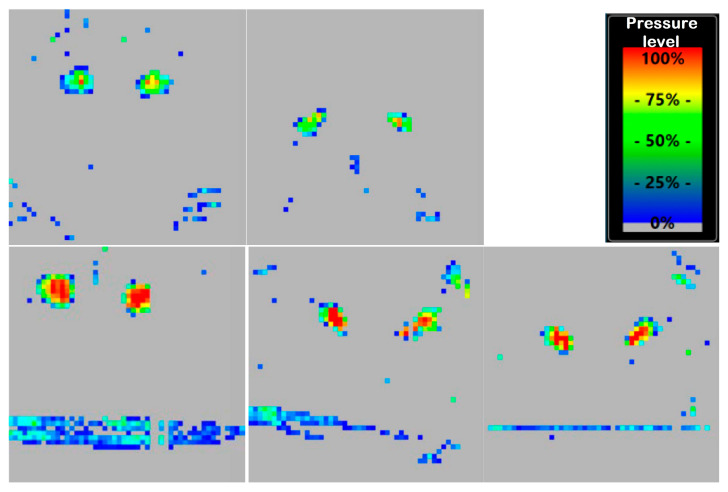
Color scale and pressure distribution on tested chairs. From left above: Chairs Nr. 1–5.

Data (pressure maps) from the initial test measurements are shown in Figure 16, where graphical outputs from the measurement program are captured on a relative color scale. The output value 255 of the A/D converter corresponds with the red color on the pressure map; according to the preliminary calibrations, it corresponds to values of pressure above 125 kPa; the lowest detectable pressure is cca. 15 kPa, marked with dark blue. The grey area corresponds to no measurable pressure. Areas of high contact pressures (over 100 kPa, pelvic bones, red areas) are captured well, and areas of lower pressures (below 50 kPa, e.g., parts of the legs, blue dots) during contact with the chair are imperceptible, or they are not captured at all, even with the largest amplification selected. This deficiency was caused by the wrong ratio of the ink blend during the manufacturing process; this was also verified by comparing the electrical resistance of the manufactured sensing plate with the corresponding “test plate”; see the discussion below.

Also visible in the images are fractures caused by bending the sensing plate over the seat of the chair. The creation of “false” pressures in the bending area is due to the sensor technology used; the material in the bending area is compressed; hence, it changes its electrical resistance. With the given production technology, this phenomenon cannot be eliminated. However, the sensing plate meets the flexibility requirements to adopt uneven surfaces and there are no breaks or “false” responses except for the bending area.

### 3.2. Testing with Modified Sensing Plate

**Figure 17:**
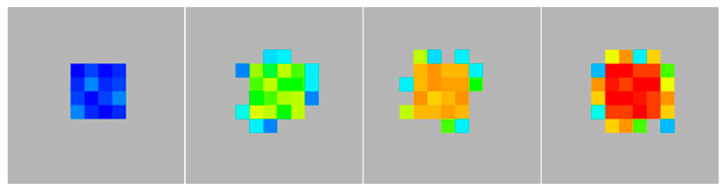
Sensor calibration with a cube. Loads 28 N, 50 N, 120 N and 200 N from left.

After manufacturing and installing a new sensor plate with the corrected ink ratio, we did a calibration of the sensor, as documented in the cutouts from the graphic outputs of the program in Figure 17. A cube with an edge length of 40 mm and a wall area of 1600 mm^2^ was placed on the sensor; a load was successively placed on the cube, which acted on the sensor with forces of 28 N (corresponding to a pressure value of 17.5 kPa), 50 N (31.25 kPa), 120 N (75 kPa) and 200 N (125 kPa), to capture the expected measurement range. The minimum value of the detectable pressure was just below 17 kPa (Figure 17 left) and the pressure values above approximately 120 kPa are displayed in red as the maximum (Figure 17 right).

**Figure 18:**
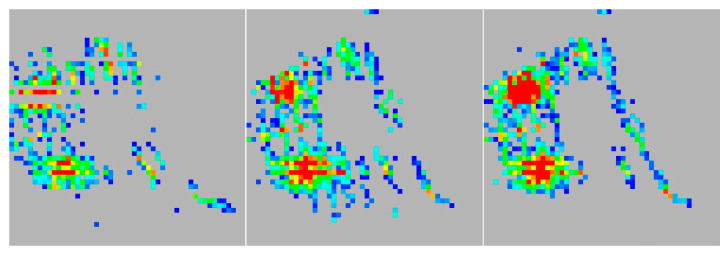
Pressure distribution with different amplification. From left: 3×—5×—10×.

Compared to the results of initial testing in the previous chapter, the sensitivity of the sensor in the real measurements improved greatly, as documented in the series of images in Figure 18. Here, the same pressure distribution on the seat of the chair (Chair Nr. 1) is captured with different gain values from the smallest (left, 3×) to the largest (right, 10×). In contrast to the initial measurements in Figure 16, significantly lower contact pressures (leg imprints) are recorded than in the previous case, when only the contact of the pelvic bones was visible.

The tactile system requires a balance between correctly capturing both lower pressure values (generally below 30 kPa) and higher pressure values above 100 kPa. Using the control program, values of zero shift and gain can be altered. The offset can be set from −8 to +8 and the gain can be set from 1× to 16×. The limiting factors are noise level and insensitivity due to overamplification. An offset value of 7 and a gain of 10× were experimentally found to be the best possible settings; these values are just below the noise limit. Higher gain values evaluate the applied pressure inaccurately; most of the compressed area would be displayed in red color and the full range of the AD converter would not be used. Despite the significant improvement of the sensitivity of the sensor, it is still not optimal in the range of lower pressures (below cca. 30 kPa); this issue should be further addressed. With a suitable combination of gain and zero shift, sufficient sensitivity of the sensor can be achieved for the given purposes.

## 4. Discussion

The general usability of the developed tactile system has been proved by the testing measurements on chair seats; however, its sensitivity in the lower pressure areas, generally below 30 kPa, may be improved. There are several technical solutions to address this issue.

**Figure 19:**
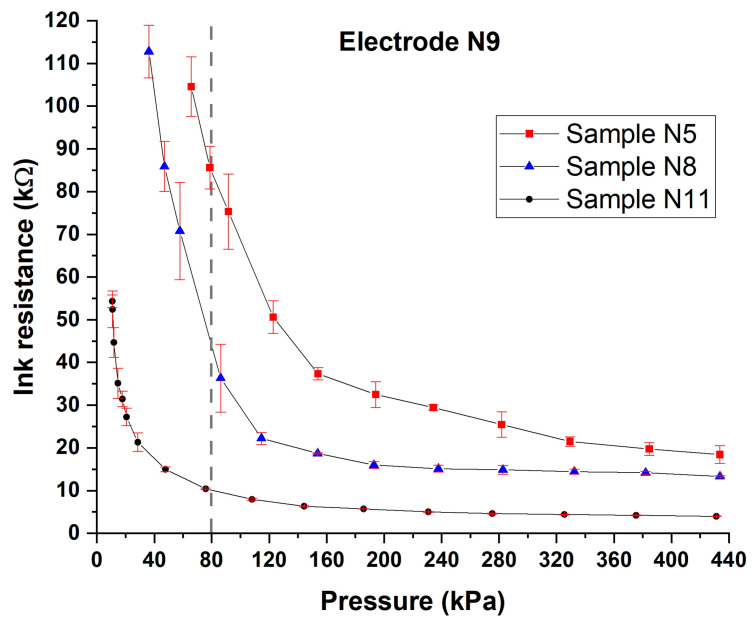
Comparing of loading characteristics. Ratio of NCI 7002 and ECI 7004 in the mixture 60:40 (N5), 50:50 (N8) and 40:60 (N11), Electrode Nr. 9.

Better sensitivity of the sensor could be achieved most easily, without changing its design, by choosing a higher ratio of the ECI 7004 conductive ink in the mixture. Under the same load, higher proportion of the conductive ink ECI 7004 in the mixture causes significant decrease in the measured electrical resistance of the ink layer. In the graphs in Figure 19, the loading characteristics labeled N5 (mixture NCI 7002 to ECI 7004 60:40); N8 (50:50) and N11 (mixture NCI 7002 to ECI 7004 40:60; the same mixture used in the sensor) are stated. By inverting the ratio of inks in the mixture (60:40 instead of 40:60 in favor of ECI 7004), the measured electrical resistance under given load (e.g., 80 kPa, dashed line) decreases about 10 times between variants N5 and N11. This subsequently causes a substantial increase in the sensitivity of the sensor in the range of lower pressures. Also, the examples demonstrated from initial “test” and subsequent “after repair” measurements (Figure 16 and Figure 18) show a significant impact of a 20 percent increase in the conductive component in the ink mixture on real measurements.

Hence, it can be expected that further improvement of the sensitivity of the sensor could be ensured by further increasing the proportion of ECI 7004 ink to values of around 70%–80%. However, increasing the proportion of conductive ink in the mixture above this value already runs into technical limitations given the measuring range. Since it is necessary to measure pressures in the whole range of at least 30–200 kPa, it may happen that while the sensor would be able to measure lower pressures in the indicated range, it would become insensitive to higher pressures; this is caused by an excessive decrease in the electrical resistance of the loaded sensor and the subsequent saturation of the material as explained in the theoretical part of this study.

Another possibility aimed at improving the sensitivity of the sensor is a partial modification of the structure. This would require printing the circular electrodes on the dielectric layer in one level (see Figure 9: (3), (4)). Due to the location of the supply wires, however, it is necessary that the wires do not cross each other. This can be achieved by double-sided printing, where the conductors for the rows will be printed on one side of the carrier layer and on the other side for the columns. However, since this technology was not available during the manufacture of the sensor, it was necessary to print the electrodes and supply wires on two dielectric layers. However, due to this, the inner and outer ring electrodes are not placed at the same level; even a difference in the order of micrometers negatively affected the ability of the sensor to capture lower pressures, since at low applied pressure, the ink layer does not completely adhere to the inner electrode through the air gap. The aforementioned deficiency can probably be completely eliminated by the appropriate design modification as discussed above.

## 5. Conclusions

We have developed a fully functional prototype of an original flexible pressure distribution tactile system that includes the sensing plate, the control electronic circuits and the control PC program. The piezoresistive transducing technology that converts the acting pressure into electric signal proved it suitability for use in such types of sensors. The main advantages are easy-to-design and easy-to-produce sensing plate by means of PCB technology and relatively less complicated electronic control circuits compared to different sensing technologies.

The active area of the sensing plate has dimensions of 50 × 50 cm with 50 × 50 sensors placed in a matrix, which corresponds to the sensor density of 1 sensor per 1 cm^2^, but the control electronics circuits are ready to process up to 80 × 80 resolution. After the preliminary evaluation of the sensor, the design with holes in the sensing plate ensures sufficient flexibility to adopt uneven surfaces. From this point of view, the tactile system is fully capable of measuring the pressure distribution on a relative 256-level scale according to the assignment.

The SITSCAN CS tactile system is primarily intended for measuring the surface distribution of pressure on the seat of a chair; however, it will be possible to use the sensor, either directly or after partial modifications, for other biomechanical applications, such as measuring the pressure under the foot to diagnose flat feet. Due to the high working frequency, which was intentionally oversized, it is also able to measure very dynamic pressure changes, e.g., in sport training analysis where it needs to be captured highly dynamic movements.

After adjusting the sensor to the measuring range and additional protection of the sensor from damage, it can also be used in other industrial applications: for example, in the automotive industry, in tire development, when measuring wiper contact pressures or optimizing the seats of cars and trucks, work machines or agricultural machines. The tactile sensor can also be inserted into the soil to determine the degree of its compaction in depth. Even in these cases, however, it would be necessary to design an optimal sensing matrix with the required resolution and sensitivity according to the respective application.

According to the initial tests of the tactile system, it captures well the pressures of cca. 30 kPa and above; below this level, its sensitivity is not as sufficient as we expected, which can be seen on the respective imprints of the chair seats. This issue can be managed by appropriate design modifications, namely modifying the ratio of the ink mixture or selecting different printing technology, i.e., double-sided printing.

The main advantage of the tactile system is its modularity, i.e., to the same control electronics and using a common PC program can be attached various sensing plates, provided that they have the maximum allowed 80 × 80 resolution. Using PCB printing technology, the manufacturing of sensing plates with different dimensions and resolutions can be relatively rapid and inexpensive.

In the future, we will continue in the enhancements of the sensor, particularly to improve its ability to measure lower pressure ranges, and we will investigate other, more flexible materials that allow the selected PCB manufacturing technology too. Further research will also aim to enable connecting several sensing plates in a sequence to create a “sensing carpet” with a length up to 2 m.

The presented tactile system SITSCAN CS can reach the properties of commercially available tactile systems with lower costs and similar technical parameters; for some parameters, such as refresh rate or possible resolution, it can even surpass them. The tactile system will undergo extensive testing by designing ergonomically shaped chairs.

## 6. Patents

Patent Nr. 309605. Pressure distribution sensing device. VOLF Jaromír; PAPEŽOVÁ Stanislava; NOVÁK Viktor; KODER Petr and SYROVÝ Tomáš. 2023. https://isdv.upv.gov.cz/doc/FullFiles/Patents/FullDocuments/309/309605.pdf (accessed on 9 March 2026).

## Figures and Tables

**Table 1:** Properties of the tested samples.

Variant Nr.	Ink Mixture Ratio 7002:7004	Ink Thickness in μm
Plate 3	40:60	8–9
Plate 4	40:60	10–12
Plate 5	40:60	24–27
Plate 6	50:50	8–9
Plate 7	50:50	10–12
Plate 8	50:50	24–27
Plate 9	60:40	8–9
Plate 10	60:40	10–12
Plate 11	60:40	24–27

## Data Availability

The raw data supporting the conclusions of this article will be made available by the authors on request.

## Abbreviations

The following abbreviations are used in this manuscript:

ADC	Analog-to-digital converter
FSR	Force-sensing resistor
MCU	Microcontroller unit
MOSFET	Metal–oxide–semiconductor field-effect transistor
PEDOT	Poly(3,4-ethylenedioxythiophene)
PET	Polyethylene terephthalate
PCB	Printed circuit board
